# Ovarian Endometriosis Accelerates Premature Ovarian Failure and Contributes to Osteoporosis and Cognitive Decline in Aging Mice

**DOI:** 10.3390/ijms26073313

**Published:** 2025-04-02

**Authors:** Lei Ge, Yali Yang, Tianxia Xiao, Yuqing Gao, Wakam Chang, Feifei Du, Ming Yu, Jian V. Zhang

**Affiliations:** 1 Center for Energy Metabolism and Reproduction, Shenzhen Institutes of Advanced Technology, Chinese Academy of Sciences, Shenzhen 518055, China; lei.ge@siat.ac.cn (L.G.); ming.yu1@siat.ac.cn (M.Y.); 2 University of Chinese Academy of Sciences, Beijing 100049, China; 3 Shenzhen Key Laboratory of Metabolic Health, Shenzhen 518000, China; 4 Department of Biomedical Sciences, Faculty of Health Sciences, University of Macau, Macau 999078, China; 5 Faculty of Pharmaceutical Sciences, Shenzhen University of Advanced Technology, Shenzhen 518028, China; 6 Sino-European Center of Biomedicine and Health, Shenzhen 518000, China

**Keywords:** ovarian endometriosis, primordial follicles activation, premature ovarian failure, ovarian reserve depletion, systemic health effects

## Abstract

Ovarian endometriosis (OEM) is a chronic inflammatory condition that impairs ovarian function. While its effects on ovarian reserve are well established, the long-term consequences of OEM on ovarian dysfunction, premature ovarian failure (POF), and systemic health, particularly in the context of accelerated aging, remain insufficiently studied. In this study, we employed an OEM mouse model and bulk RNA sequencing to investigate the underlying mechanisms. Our results show that OEM accelerates primordial follicle depletion and upregulates mTOR signaling pathway gene expression, along with mechanical stress and paracrine signaling via the Hippo and Myc pathways. OEM also induces irregular estrus and ovarian fibrosis in aging mice, decreases serum AMH levels, and increases FSH levels. Systemically, elevated serum IgG levels contribute to osteoporosis and cognitive decline, both linked to ovarian dysfunction and POF in OEM. These findings elucidate the mechanisms driving premature ovarian reserve depletion in OEM and highlight its broader systemic effects. This study emphasizes the importance of monitoring ovarian health and ectopic tissue to safeguard ovarian reserves and mitigate long-term risks such as osteoporosis and cognitive decline.

## 1. Introduction

Endometriosis (EM) is a prevalent gynecological disorder affecting 5–10% of women of reproductive age globally, often causing infertility and chronic pain [1]. However, due to nonspecific symptoms and diagnostic challenges, EM is frequently diagnosed late, leading to prolonged ovarian damage from ectopic lesions. This damage compromises fertility and may accelerate ovarian function decline [2,3]. EM impairs fertility through several mechanisms, including tubal adhesions, abnormal activation of primordial follicles, and decreased oocyte quality [4,5,6].

EM is classified into three subtypes, with ovarian endometriosis (OEM) being the most common and destructive, characterized by the formation of ectopic cysts within the ovaries. Unlike other forms of EM, OEM exerts the most significant impact on reproductive function [7]. Studies indicate that compared to women with healthy ovaries or other benign ovarian cysts, those with OEM show markedly lower levels of anti-Müllerian hormone (AMH) [8,9], suggesting that OEM may reduce ovarian reserve by disrupting the ovarian microenvironment, further impairing fertility [7,10]. However, despite its clear effects on the ovarian reserve, few studies have examined whether OEM contributes to the long-term decline in ovarian function, premature ovarian failure (POF), or accelerated perimenopausal changes.

Primordial follicles, the basic reproductive units of the ovary, are maintained in a dormant state and activated in a regulated manner to preserve the ovarian reserve and reproductive capacity. Our previous studies show that OEM disrupts this balance by damaging oocytes via the TZP-mediated communication between granulosa cells and oocytes [6]. This raises the hypothesis that OEM may accelerate ovarian reserve depletion through inflammation and oxidative stress, leading to premature ovarian failure and the early onset of perimenopause.

While many studies have focused on the immediate impact of OEM on the ovarian reserve, there is a lack of research into its long-term consequences, especially regarding early perimenopause. The early onset of perimenopause significantly shortens the reproductive lifespan and increases the risk of serious conditions like osteoporosis [11], cognitive decline [12], and cardiovascular disease [13], which severely affect women’s health and quality of life. Understanding the mechanisms by which OEM accelerates ovarian aging and perimenopausal changes is therefore crucial for developing effective clinical strategies and interventions.

This study aims to explore the mechanisms by which OEM accelerates ovarian aging and perimenopausal changes, with a focus on the systemic health implications, using mouse models. Our findings show that OEM accelerates ovarian aging, with premature depletion of the primordial follicle pool, disrupted sex hormone levels, and subsequent effects on osteoporosis and cognitive function. This research provides critical insights into the long-term health consequences of OEM, offering a theoretical basis for improved clinical management of women with EM. By elucidating the molecular pathways linking OEM to accelerated ovarian aging and systemic health risks, this study could inform future interventions aimed at preventing early perimenopause and its associated diseases, thus improving women’s overall health outcomes.

## 2. Results

### 2.1. Abnormal Activation of Primordial Follicles in OEM

**Figure 1:**
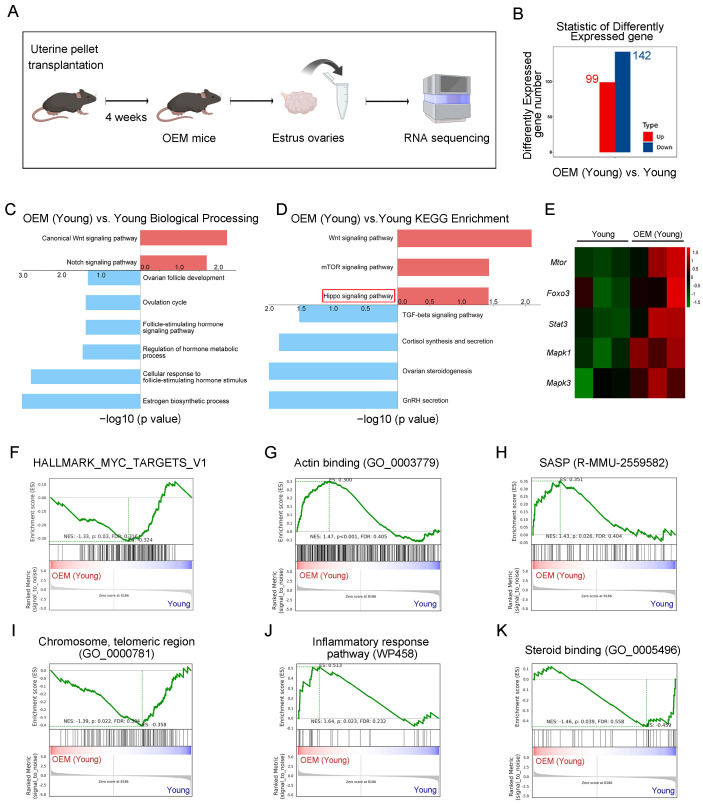
RNA sequencing and pathway analysis of OEM in mice. (**A**) Experimental overview: 9-week-old OEM mice underwent uterine pellet transplantation, and ovarian tissues were collected 4 weeks post-surgery for RNA sequencing. (**B**) Differentially expressed genes (DEGs) between OEM (young) and young control mice. (**C**,**D**) Gene ontology (GO) and Kyoto Encyclopedia of Genes and Genomes (KEGG) enrichment analysis showing significant biological processes and pathways associated with the DEGs in OEM (young) and young mice. The plots highlight the top processes with significant changes (*p* < 0.05, |log2 fold change| > 1.5). (**E**) Heatmap shows key genes expression changes associated with primordial follicle activation. (**F**–**K**) Gene set enrichment analysis (GSEA) revealing significant alterations in gene sets related to MYC signaling (**F**), actin binding (**G**), senescence-associated secretory phenotype (SASP) (**H**), chromosome telomeric regions (**I**), inflammatory response (**J**), and steroid binding proteins (**K**). Enrichment scores (NES) represent the degree of pathway activation or inhibition, with significant results denoted by a *p*-value < 0.05.

Our previous study demonstrated that OEM impairs oocyte quality by disrupting communication between oocytes and granulosa cells, which leads to an increased number of atretic follicles and potentially abnormal activation of primordial follicles [6]. To further investigate the effects of OEM on ovarian function, we performed bulk RNA sequencing on ovarian tissues from OEM mice four weeks post-surgery (Figure 1A). Differential gene expression analysis identified 99 upregulated genes and 142 downregulated genes in the OEM group compared to controls (Figure 1B).

Gene ontology (GO) enrichment analysis revealed a significant upregulation of the Wnt and Notch signaling pathways (*p* < 0.05, |log2 fold change| > 1.5), indicating activation of pathways typically involved in follicular activation and cellular proliferation (Figure 1C). In contrast, ovarian follicle development and hormone synthesis were downregulated, pointing to disruptions in normal ovarian function (Figure 1C). Pathway enrichment analysis (KEGG) further highlighted an increase in the Wnt, mTOR, and Hippo signaling pathways, while TGF-beta and sex hormone pathways were significantly downregulated (Figure 1D).

The heatmap illustrates gene expression changes associated with primordial follicle activation in the OEM model (Figure 1E). Gene set enrichment analysis (GSEA) highlighted a significant decrease in MYC signaling (Figure 1F) and an increase in actin-binding proteins, which may indicate changes in cytoskeletal dynamics (Figure 1G). In the OEM group, senescence-associated secretory phenotype (SASP) markers were elevated, indicating the mechanical compression of ovarian tissue (Figure 1H). Additionally, a decrease in chromosome telomeric regions suggests that OEM may induce chromosomal instability and accelerate cellular senescence (Figure 1I). Furthermore, there was an increase in genes associated with the inflammatory response and steroid binding proteins, indicating that OEM triggers an inflammatory cascade that may contribute to ovarian tissue damage and hormonal dysregulation (Figure 1J,K). These findings suggest that OEM induces abnormal activation of primordial follicles, contributing to premature ovarian aging and dysfunction.

### 2.2. Estrous Cycle Disruption and Ovarian Reduction in Aging Mice with OEM

**Figure 2:**
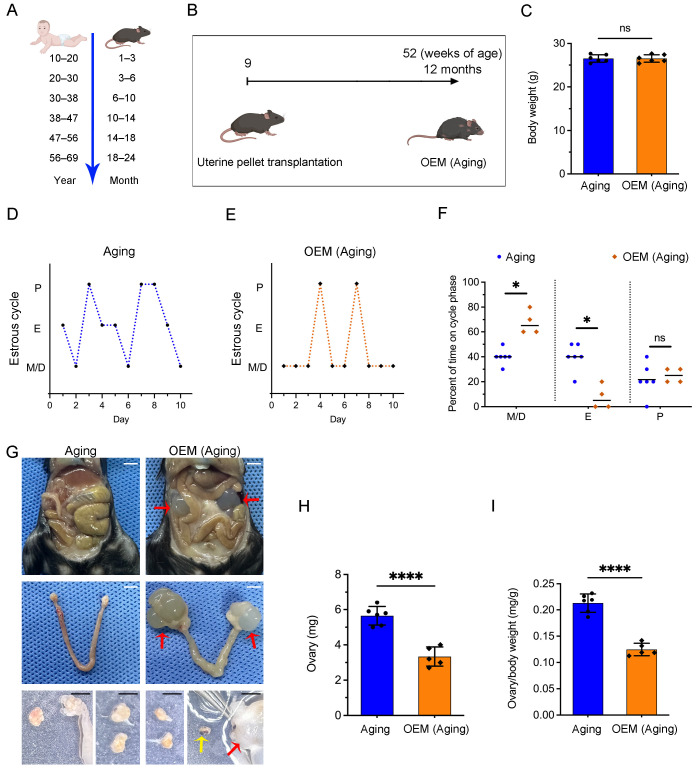
Effects of OEM on estrous cyclicity and ovarian health in aging mice. (**A**) Analogical time axis comparing the aging process in mice and humans. Twelve months of age in mice corresponds to perimenopause in humans. (**B**) Experimental timeline for the OEM mouse model. Nine-week-old OEM mice underwent uterine pellet transplantation, and 12-month-old mice were used for subsequent testing. (**C**) Comparison of body weight between OEM and aging control mice. (**D**) and (**E**) Representative estrous cyclicity of aging and OEM (aging) mice over 10 consecutive days. The phases are as follows: M/D, metestrus/diestrus; P, proestrus; E, estrus. (**F**) Quantitative analysis of estrous cyclicity in aging (12 months) mice from aging and OEM (aging). Scatterplot displaying the percentage of time spent in each estrous phase for aging and OEM (aging). (**G**) Representative images of single or multiple cystic lesions observed in the ovaries of the OEM (aging) group (red arrows), with multiple hemorrhagic cysts (HC) present in the OEM (aging) ovaries (yellow arrow). Scale bar: 5 mm. (**H**,**I**) Comparison of ovarian weight and ovarian coefficient between the aging OEM and control groups. Each dot represents an individual mouse. * *p* < 0.05 and **** *p* < 0.0001. Not significant (ns) at *p* ≥ 0.05.

To examine whether OEM accelerates premature ovarian failure (POF) through the early depletion of primordial follicles, we established a model using 9-week-old mice, which were then assessed at 12 months of age. Small changes in ovarian function during this period can be more readily detected through comparison. This corresponds to a human age equivalent of 40–50 years, a period during which reproductive capacity typically declines (Figure 2A,B). No significant differences in body weight were observed between 12-month-old OEM and control mice (Figure 2C).

Estrous cycle analysis over a 10-day period revealed that the estrous cycle of aging control mice exhibited typical fluctuations, with noticeable estrus phases during the observation period (Figure 2D). In contrast, OEM mice exhibited disrupted cycles, with extended durations in the metestrus (M) and diestrus (D) phases and a marked absence of estrus (E) (Figure 2E). Quantitative analysis confirmed a significant increase in the duration of the M/D phases in OEM-treated mice compared to controls, while the duration of the E phase decreased from 40% in controls to just 8% in the OEM group (Figure 2F).

At 12 months, mice were euthanized, and endometrial lesions were examined. Cystic lesions were found surrounding the bilateral ovarian tissues in all mice from the OEM group (Figure 2G). Additionally, a significant reduction in ovarian weight and ovarian coefficient was observed in OEM-treated mice compared to aging controls (Figure 2H,I). These results indicate that OEM leads to estrous cycle disruption, ovarian reduction, and the development of POF in aging mice.

### 2.3. Premature Exhaustion of Primordial Follicles and Accumulation of Ovarian Fibrosis in OEM

**Figure 3:**
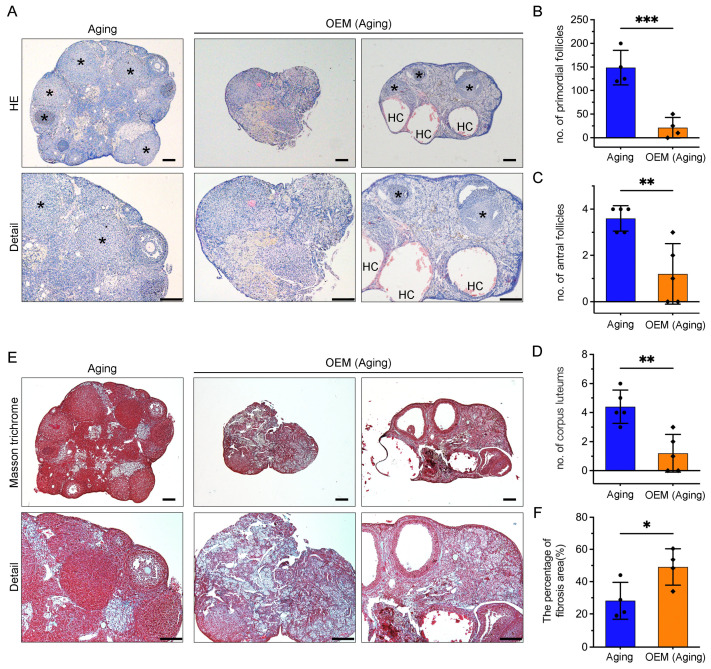
Histological analysis of ovarian structure and fibrosis in OEM. (**A**) Hematoxylin and eosin staining of representative ovaries. “HC” indicates hemorrhagic cysts, and corpora lutea are marked with asterisks. Scale bar: 200 µm. (**B**–**D**) Quantitative analysis of primordial follicles, antral follicles, and corpora lutea in ovarian sections. (**E**) Ovarian fibrosis measured by Masson’s trichrome staining to detect collagen. Scale bar: 200 µm. (**F**) Comparison of the percentage of fibrosis area between OEM (aging) and aging control groups. Each dot represents an individual mouse. * *p* < 0.05, ** *p* < 0.01 and *** *p* < 0.001.

To assess the impact of OEM on follicular development, we performed serial sections of ovaries from 12-month-old mice. Histological examination revealed corpora lutea in the aging control group (Figure 3A, left asterisk). In contrast, two distinct patterns were observed in the OEM group: one group exhibited complete depletion of follicles with no follicular structures remaining (Figure 3A, middle), while the other group showed a small number of corpora lutea, the absence of antral or preovulatory follicles, and the presence of hemorrhagic cysts (HC) (Figure 3A, right). Statistical analysis confirmed a significant reduction in the number of primordial follicles (Figure 3B) and antral follicles (Figure 3C) in the OEM group compared to controls. Additionally, the number of corpora lutea was significantly reduced (Figure 3D), further indicating that OEM accelerates the depletion of ovarian follicles.

As aging is associated with collagen accumulation, which may inhibit follicular growth and ovulation, we examined ovarian fibrosis using Masson’s trichrome staining (Figure 3E). The results showed that OEM significantly exacerbated the development of ovarian fibrosis (Figure 3F). This increased fibrosis likely exacerbates follicular depletion by altering the ovarian microenvironment, disrupting hormonal and cellular signaling, restricting blood flow, and increasing inflammation, further accelerating the onset of POF.

### 2.4. Premature Hormonal Aging and Increased IgG Levels in OEM

**Figure 4:**
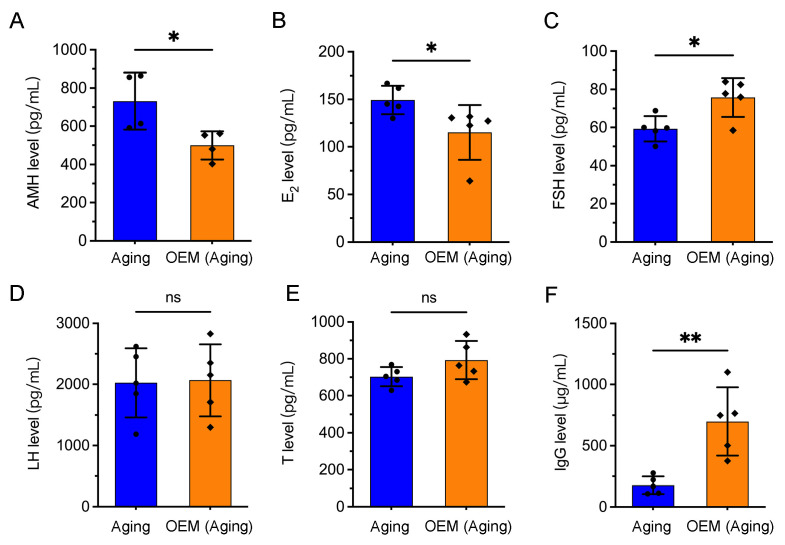
Serum hormone and IgG levels in aging and OEM Mice. Serum levels of anti-Müllerian hormone (AMH) (**A**), E_2_ (**B**), follicle-stimulating hormone (FSH) (**C**), luteinizing hormone (LH) (**D**), testosterone (T) (**E**), and immunoglobulin G (IgG) (**F**) were measured by ELISA. Each dot represents an individual mouse. * *p* < 0.05, ** *p* < 0.01. Not significant (ns) at *p* ≥ 0.05.

Alterations in ovarian follicular composition and premature follicular depletion can impair the production and secretion of key female hormones, with consequences for various physiological systems [14,15]. To investigate the hormonal changes associated with OEM, we measured hormone levels in the serum of aged mice. Anti-Müllerian hormone (AMH), a marker of the ovarian reserve, was significantly reduced in OEM-aged mice compared to controls (Figure 4A). Similarly, E_2_ levels were significantly decreased (Figure 4B), which aligns with the depletion of ovarian follicles and reduced ovarian function. In contrast, follicle-stimulating hormone (FSH) levels, a key indicator of premature ovarian aging, were significantly elevated in OEM-aged mice relative to controls (Figure 4C), suggesting an accelerated decline in ovarian function. However, no significant differences were observed in luteinizing hormone (LH) or testosterone (T) levels between the groups (Figure 4D,E).

Recent studies have linked elevated immunoglobulin G (IgG) levels to aging-related biological changes [16]. Consistent with this, we observed a significant increase in serum IgG levels in OEM-aged mice compared to aging controls (Figure 4F). Together, these findings suggest that OEM contributes to premature hormonal aging and increased IgG levels, which may serve as indicators of broader systemic effects associated with ovarian dysfunction.

### 2.5. Premature Osteoporosis Induced by OEM in Perimenopausal Women

**Figure 5:**
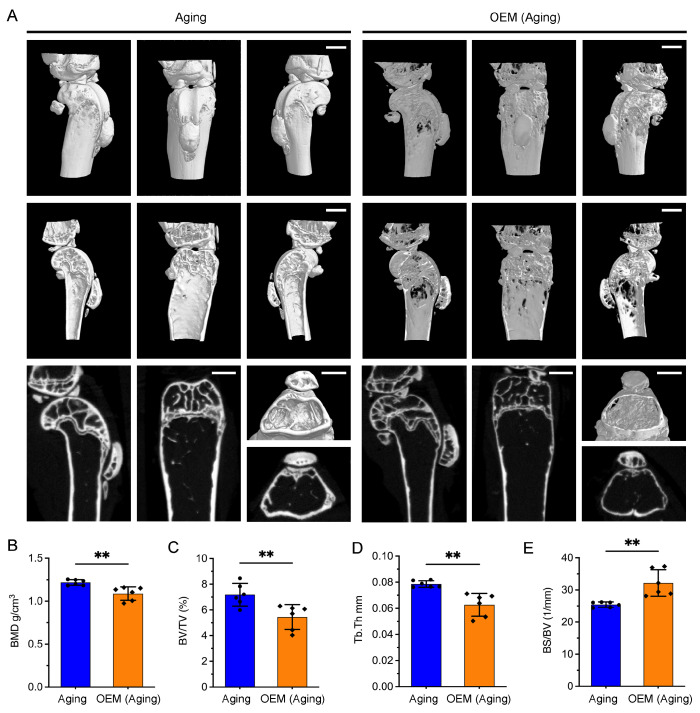
Micro-CT analysis of trabecular bone structure in the distal femur of aging and OEM mice. (**A**) Representative 3D images illustrating the trabecular microarchitecture in the distal femoral metaphysis of OEM and aging controls. Scale bar, 1 mm. (**B**–**E**) Microstructural parameters of the distal femoral trabecula, as analyzed from micro-CT scanning data: (**B**) bone mineral density (BMD); (**C**) fractional bone volume (BV/TV, %); (**D**) trabecular thickness (Tb.Th, mm); (**E**) Bone surface-to-bone volume ratio (BS/BV, 1/mm). Each dot represents an individual mouse. ** *p* < 0.01.

OEM accelerates hormonal aging, which may contribute to osteoporosis, a condition more prevalent in perimenopausal women and associated with an increased risk of fractures [12,17]. We performed micro-CT scanning of the distal femoral trabecular bone in aging OEM mice. Three-dimensional reconstruction images revealed distinct microstructural differences between the OEM and control groups (Figure 5A).

Micro-CT analysis showed a significant reduction in trabecular bone mineral density (BMD) in OEM mice compared to controls (Figure 5B), indicating weakened bone strength and an increased fracture risk. Additionally, fractional bone volume (BV/TV) was markedly decreased (Figure 5C), and trabecular thickness (Tb.Th) was reduced (Figure 5D), both of which are critical indicators of bone integrity. The increased bone surface-to-bone volume ratio (BS/BV) in OEM-treated mice (Figure 5E) suggests disrupted bone remodeling, further contributing to bone brittleness. These findings indicate that OEM accelerates bone loss and disrupts the microstructural integrity of trabecular bone, contributing to premature osteoporosis in aging mice.

### 2.6. Acceleration of Anxiety and Cognitive Impairment in Aging Mice Induced by OEM

**Figure 6:**
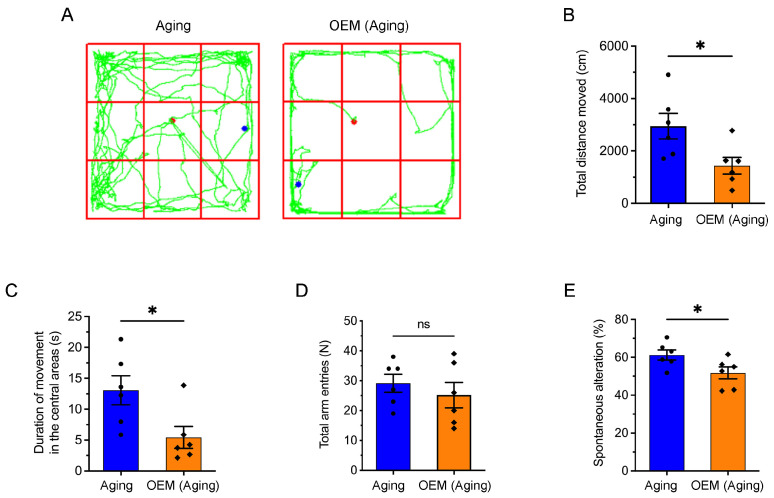
Behavioral assessment of anxiety and cognitive function in aging and OEM mice. (**A**) Representative motion tracks from the open field test showing the movement patterns of aging (Left) and OEM (Right) mice. (**B**,**C**) Total distance moved (**B**) and time spent in the central area (**C**) of the open field test. (**D**,**E**) Total arm entries (**D**) and percentage of spontaneous alternations (**E**) in the Y-maze task. Each dot represents an individual mouse. * *p* < 0.05. Not significant (ns) at *p* ≥ 0.05.

Clinical studies have shown that perimenopausal and postmenopausal women are at a higher risk for cognitive disorders, including Alzheimer’s disease (AD) [18]. Moreover, elevated FSH levels in perimenopausal women have been linked to an increased incidence of AD [19]. To explore whether OEM accelerates anxiety and cognitive impairment in aging mice, we assessed the movement and cognitive abilities of mice using the open field test (OFT) (Figure 6A).

In the OFT, we observed that the total distance traveled by OEM aging mice was significantly reduced (Figure 6B), as well as the time spent in the center of the arena, which suggests increased anxiety (Figure 6C). Further cognitive assessment using the Y-maze revealed no significant change in the total number of wall entries in OEM aging mice (Figure 6D), but a significant reduction in the number of spontaneous arm entries (Figure 6E), a measure commonly used to assess working memory and exploratory behavior. These results indicate that OEM accelerates the onset of both anxiety and cognitive impairment in aging mice. Given that these behaviors closely resemble symptoms observed in clinical conditions such as AD, these findings highlight the potential link between OEM and cognitive decline in aging women, particularly in the context of perimenopausal hormonal changes.

## 3. Discussion

OEM is a chronic inflammatory condition that impairs ovarian function and has broad systemic consequences. In our previous study, we demonstrated that OEM reduces oocyte quality and increases atretic follicles, suggesting that long-term exposure accelerates premature ovarian failure (POF) in mice. Building on this, our current study further explores how OEM accelerates primordial follicle depletion, leading to POF, and contributes to hormonal disturbances, osteoporosis, and cognitive decline in aging mice. These findings highlight the broader impact of OEM on female aging and the urgent need for early intervention to prevent further systemic damage.

Our results indicate that the gene expression levels of the mTOR signaling pathway are upregulated, which may promote primordial follicle activation through the PI3K-PTEN-Akt-Foxo3 cascade signaling pathway, thereby affecting the maintenance of the ovarian reserve. This finding aligns with prior research and supports the notion that ectopic tissue in OEM models contributes to ovarian dysfunction [4,20]. Interestingly, similar activation has been reported in peritoneal EM, suggesting a shared mechanism for primordial follicle activation across both forms of EM. These findings reinforce the idea that primordial follicle activation is a key feature of both conditions [5,21,22].

Unlike other forms of EM, OEM exerts a direct mechanical effect on the ovaries due to its proximity and invasion of ovarian tissue [23,24]. Our study identifies alterations in the expression of genes associated with the Hippo signaling pathway, where physical compression induces local mechanical stress, leading to increased actin levels. This, combined with downregulated Myc pathway expression, may attenuate its inhibitory effect on primordial follicle activation, thereby further promoting follicle activation. These findings highlight the synergistic effect of mechanical stress and paracrine signaling on ovarian dysfunction in OEM. We also observed an increase in the SASP and telomere instability, which suggests that OEM-induced mechanical stress on the ovary leads to an upregulation of SASP-related genes and affects telomere integrity [25,26].

The overactivation and subsequent depletion of primordial follicles lead to POF. Based on our findings, early intervention to remove ectopic tissue is crucial in clinical practice to prevent irreversible fertility damage. However, surgery is generally not recommended for cysts smaller than 4 cm due to their proximity to the ovaries [27], as surgical intervention may exacerbate follicle activation and further damage ovarian tissue [28]. Close monitoring of ectopic tissue growth and periodic evaluations are essential to determine when surgical intervention is warranted.

Under the influence of long-term OEM damage, we observed POF, accompanied by a significant reduction in the number of antral follicles. This finding correlates with the low levels of E_2_ we detected, as E_2_ is primarily secreted by granulosa cells in the follicles [29]. In OEM-aged mice, some ovaries completely lacked corpora lutea, suggesting a failure in ovulation. This also explains why some OEM-aged mice never exhibited estrus (E) in the estrous cycle assay. The ovary is a highly dynamic organ, with primordial follicles being activated in the cortical region before migrating to the medullary region for further development [30]. However, excessive ovarian fibrosis disrupts this process by impeding follicular migration and growth, thereby accelerating ovarian aging [31]. Consistent with this, we observed increased ovarian fibrosis in OEM-aged mice, in line with previous findings in young OEM models [32]. Age-associated ovarian fibrosis has been shown to progress with advancing reproductive age, and its attenuation has been reported to extend reproductive lifespan in aged mice [31,33]. Therefore, targeting ovarian fibrosis to enhance reproductive capacity in OEM mice may represent a promising therapeutic strategy. While our study revealed robust differences across multiple physiological and histological parameters between aged and OEM-aged mice, we acknowledge that several outcomes-such as AMH, E_2_, and the ovarian fibrosis area did not achieve 80% statistical power due to limited sample sizes. Despite this, the observed trends are highly consistent with prior studies and biologically plausible within the context of known OEM pathophysiology. Future investigations with larger cohorts and longitudinal designs will be valuable for validating these findings and enhancing reproducibility.

Anti-Müllerian hormone (AMH), a commonly used biomarker for assessing the ovarian reserve [34,35], typically decreases after OEM surgery [36,37,38]. However, some studies report no significant change in antral follicle count (AFC), suggesting potential discrepancies in evaluating the ovarian reserve [39]. A long-term study by Ferrero et al. on OEM patients with postoperative recurrence found that AMH and AFC levels only decreased in the early postoperative period, and no significant differences were observed from the control group before the second surgery [40]. This suggests that AMH may not be a reliable long-term marker of the ovarian reserve in cases of abnormal primordial follicle activation. Due to the temporary activation of primordial follicles after surgery, follicle activation normalizes as the ectopic tissue is removed, thus reducing the number of growing follicles and lowering AMH levels. Our findings from aging OEM mice highlight the need for additional evaluation methods beyond AMH. Clinicians must carefully consider the balance between surgical intervention, which may cause damage to the primordial follicle reserve, and conservative treatment, which may allow continued loss of primordial follicles. Further studies are needed to establish better criteria for clinical decision-making and refine treatment guidelines for EM.

Our results reinforce the idea that OEM-induced POF has significant systemic effects, notably on bone health, with elevated FSH levels contributing to bone loss and increased fracture risk, consistent with clinical findings in perimenopausal and postmenopausal women [41,42]. Additionally, recent studies have associated elevated IgG levels with aging-related tissue dysfunction, including fibrosis and metabolic deterioration [16,43]. In our study, we found significantly higher IgG levels in OEM-aged mice, suggesting a potential association between IgG elevation and aging-related ovarian dysfunction. Given these findings, monitoring IgG levels in combination with AMH may provide insights into both ovarian function and broader aging processes in OEM patients. Considering the impact of OEM on quality of life—including associations with pain, mental health challenges, and metabolic dysfunction—early intervention and long-term patient monitoring are critical for preserving fertility and overall health [44].

Our study did not involve the surgical removal of ectopic tissue in the animal model, as the primary aim was to explore the long-term effects of OEM on ovarian function and its associated health risks. While clinical studies require long-term follow-up to confirm these effects, our OEM mouse model allows for the accelerated observation of primordial follicle depletion, a process that would take decades to manifest in human studies. Although the OEM mouse model provides valuable insights, translating these findings to human patients requires caution due to differences in hormonal regulation, immune responses, and tissue remodeling. The lack of long-term clinical follow-up data on OEM patients remains a critical gap in understanding its full impact on female reproductive health. To bridge this gap, future research should integrate findings from both mouse model and longitudinal patient studies to provide a more comprehensive perspective on disease progression and potential therapeutic strategies.

In conclusion, this study highlights the complex and systemic nature of OEM, emphasizing its role in accelerating primordial follicle depletion, leading to ovarian failure, and contributing to osteoporosis and cognitive decline. Given these findings, early intervention and personalized clinical strategies are critical to preserve ovarian function and mitigate long-term health risks. Further research, including targeted therapies and novel biomarkers, is essential to improving outcomes for patients with EM.

## 4. Materials and Methods

### 4.1. Animals

Eight-week-old female C57BL/6J mice were sourced from the Guangdong Medical Laboratory Animal Center. All animal care procedures followed the Guidelines for the Care and Use of Laboratory Animals in Guangdong Province, and the experimental protocol was approved by the Animal Research Ethics Committee of the Shenzhen Institute of Advanced Technology, Chinese Academy of Sciences (Approval Number: SIAT-IACUC-200313-YYS-YM-A1105).

The murine OEM model was established using a method adapted from Hayashi’s protocol and our previous study [6,32], achieving a 100% success rate. Briefly, six 9-week-old female mice were randomly assigned to either the young group or the young (OEM) group. The young group underwent sham surgery, which involved anesthetizing the back, making an incision, clamping the ovarian fat, injecting PBS into the bursal cavity, and then suturing the wound. The young (OEM) group underwent the same procedure, except donor uterine pellets were transplanted to encapsulate the ovaries.

After surgery, both groups were housed under identical SPF-grade conditions, with free access to food and water. Ovarian transcriptome sequencing was performed four weeks post-surgery, while subsequent experiments were conducted on 52-week-old mice.

### 4.2. RNA Sequencing and Analysis

Nine-week-old female mice were used to induce OEM, and ovaries were harvested at 13 weeks for bulk RNA sequencing. Total RNA was extracted from ovarian tissues using TRIzol (Takara, Beijing, China) for RNA-seq, with three biological replicates per group. The RNA samples were sent to OE Biotechnology LTD for sequencing (Shanghai, China). The sequencing process, including library preparation and platform selection, was conducted by OE Biotechnology following their standard protocols. Gene expression levels were quantified using the FPKM method for visualization. Differentially expressed genes (DEGs) were identified using DESeq2 version 1.22.2 based on raw counts, with Benjamini–Hochberg (BH) FDR correction applied to adjust *p*-values. A gene was considered significantly differentially expressed if adjusted *p*-value (FDR) < 0.05 and |log2 fold change| ≥ 1. KEGG pathway analysis and gene set enrichment analysis (GSEA) version 0.2.31 were performed using publicly available databases. GSEA was conducted using a pre-ranked gene list based on log2 fold changes, with multiple testing correction applied using the default FDR method embedded in the GSEA algorithm, as described by Subramanian et al., PNAS, 2005 [45].

### 4.3. Estrous Cycle Detection

Estrous cycle detection was performed on 12-month-old control and OEM mice. For 10 consecutive days, at 9 a.m. daily, a sterile cotton swab moistened with physiological saline was used to collect vaginal secretions. The sample was applied to a glass slide, air-dried, and stained with hematoxylin for 5 min. Samples were then examined under a microscope, and the estrous cycle stages were determined based on cell morphology, nuclear presence, and the proportion of keratinized cells. The stages were classified as proestrus (P), estrus (E), metestrus (M), and diestrus (D).

### 4.4. Histological Staining and Follicle Counting

Ovaries were fixed overnight in 4% paraformaldehyde (PFA), embedded in paraffin, and sectioned at 5 µm intervals. Sections were stained with hematoxylin and eosin (H&E). Follicles at different developmental stages were counted in every fifth section (spaced 25 µm apart). Total follicle counts were estimated by multiplying the number of follicles in the sections by five. Ovarian fibrosis was assessed using Masson’s trichrome staining on the largest cross-sections of each ovary, following the methodology described in a previous study [32].

### 4.5. Serum Hormone Measurement

The serum levels of AMH (SP14519), E_2_ (SP14086), FSH (SP14079), LH (SP14102), and T (SP14088) in mice were evaluated using ELISA kits (Spbio, Wuhan, China) according to the manufacturer’s instructions. The absorbance was measured at 450 nm. A standard curve was constructed using known concentrations of standard samples, and the samples were measured according to the recommended dilution ratios. The serum IgG level was evaluated using an ELISA kit (Starter, S0C3008, Hangzhou, China), with absorbance measured at 450 nm.

### 4.6. Micro-Computed Tomography (Micro-CT) Analysis

Micro-CT imaging was used to evaluate the trabecular and cortical bone microarchitecture. Tibiae from each group (n = 4 per group) were harvested, rinsed in PBS, and fixed in 4% PFA. Bone scanning was conducted using a ZEISS Xradia Versa XRM-520 high-resolution CT system (Carl ZEISS XRM, Pleasanton, CA, USA) [46,47], with a slice thickness of 9 µm and consistent reconstruction parameters across all scans. Specifically, the axial images obtained were imported into DataViewer software version 1.5.2.4 for reconstruction and reading. We then used the CTVol program (Skyscan) to generate 3D images. For BMD measurement and further analysis, the CTAn software v.1.18 (Skyscan) was employed. Quantitative morphometric analysis, including trabecular number (Tb.N), trabecular thickness (Tb.Th), trabecular separation (Tb.Sp), bone volume-to-tissue volume ratio (BV/TV), and bone surface-to-bone volume ratio (BS/BV), was performed using the system’s software.

### 4.7. Open-Field Test

Exploratory activity and anxiety-like behavior were assessed using an open-field test (50 cm × 50 cm × 50 cm). Each mouse was placed in a corner of the arena, with the central zone defined as a 10 cm square in the center. The total distance traveled, time spent in the central area, movement time, and average speed were recorded over a 10 min period using a video tracking system (EthoVision XT, Noldus Information Technology, Wageningen, The Netherlands) [48].

### 4.8. Y-Maze Test for Spontaneous Alternation

Spontaneous alternation was evaluated using a black, symmetrical Y-maze with three arms (39.5 cm in length, 8 cm in width, and 20 cm in height) arranged at 120° angles. Mice were placed at the distal end of arm A and allowed to explore the maze for 8 min, as described by Carroll et al. [49]. Movement was recorded via an overhead camera for analysis. Spontaneous alternation was characterized by the mice making three consecutive entries into distinct arms, such as the sequences ABC, BCA, and CAB. The percentage of spontaneous alternation was determined using the following formula: spontaneous alternation number/(total number of entries − 2) × 100%. To maintain cleanliness between trials, the maze was wiped down with 75% ethanol after each use.

### 4.9. Statistical Analysis

To ensure sufficient statistical power for detecting biologically meaningful differences, we conducted a power analysis using G*Power for independent *t*-tests, assuming α = 0.05 and power = 80%. Data are presented as mean ± standard deviation (SD). Statistical significance was determined based on at least three independent biological replicates. Staining experiments were repeated at least three times, with representative micrographs shown. Statistical significance is indicated as * *p* < 0.05, ** *p* < 0.01, and *** *p* < 0.001. Not significant (ns) at *p* ≥ 0.05.

## Data Availability

The original contributions presented in this study are included in the article. Further inquiries can be directed to the corresponding author.
